# Neuroimaging in schizophrenia: From group-average abnormalities to individualised circuit models

**DOI:** 10.1177/00368504261465544

**Published:** 2026-07-02

**Authors:** Wesley Pyke, Sukhwinder S. Shergill

**Affiliations:** 1 School of Psychology, University of Kent, Canterbury, UK; 2 583155 Kent and Medway Medical School, Canterbury, UK; 3 Department of Psychosis Studies, Institute of Psychiatry, Psychology and Neuroscience (IoPPN), King’s College London, London, UK; 4 Kent and Medway Mental Health and Community Services, Maidstone, UK

**Keywords:** schizophrenia, neuroimaging, network dysconnectivity, thalamocortical circuits, dopamine–glutamate interaction, normative modelling, precision psychiatry

## Abstract

This narrative review synthesises advances in multimodal neuroimaging that, over the past five years, have substantially refined models of schizophrenia pathophysiology. Converging evidence from structural, diffusion, functional, molecular, and computational studies challenges static, regionally focal, or single-neurotransmitter accounts of the disorder. Instead, contemporary findings support a framework in which schizophrenia reflects heterogeneous, developmentally anchored deviations in distributed neural circuits that manifest as large-scale network instability. Structural and diffusion imaging reveal individually variable patterns of cortical, white matter, and thalamic subnuclei alterations. Functional MRI demonstrates impaired regulation across salience, default mode, executive, and thalamocortical systems, while positron emission tomography and magnetic resonance spectroscopy implicate subregion-specific dopaminergic and glutamatergic abnormalities. Computational modelling further indicates that these multilevel disturbances may converge on altered synaptic gain and excitation–inhibition imbalance within cortical microcircuits, providing a mechanistic substrate for systems-level dysconnectivity. Importantly, heterogeneity is not incidental but central: normative modelling and biologically informed subgrouping demonstrate that while group-level effects are robust, the anatomical and neurochemical loci of deviation vary substantially across individuals. We propose that shared network instability may arise from diverse developmental perturbations across cortico–striato–thalamo–cortical circuits. Future progress will depend on longitudinal, harmonised, and multimodal study designs capable of modelling individual trajectories across risk stages and treatment exposure. Conceptualising schizophrenia as a dynamically evolving circuit disorder therefore offers an integrative framework that bridges molecular dysfunction and clinical expression and provides a roadmap for mechanism-informed stratification, while clinical translation remains a longer-term objective.

## Introduction

Schizophrenia is a severe and heterogeneous psychotic disorder within a broader schizophrenia spectrum that includes schizotypal personality disorder, delusional disorder, brief psychotic disorder, and schizoaffective disorder.^1^ Its clinical presentation spans positive symptoms such as hallucinations and delusions, negative symptoms including apathy and social withdrawal, and pervasive cognitive impairments affecting attention, working memory, and executive functioning.^2^ These symptom domains frequently disrupt education, employment, relationships, and independent living, contributing to substantial personal and societal burden.^3,4^

Despite decades of research, however, schizophrenia remains without a definitive biological marker. Neuroimaging studies consistently demonstrate structural, functional, and neurochemical alterations, yet no single anatomical region or molecular pathway fully accounts for the disorder. Group-level differences are reproducible, but individual variability is striking. This heterogeneity has complicated efforts to define schizophrenia as a focal brain disease and has instead prompted a reconceptualisation of the disorder as involving distributed neural systems.

Historically, explanatory models centred on the classical dopamine hypothesis, attributing psychotic symptoms to excessive dopaminergic transmission.^5^ While this framework remains influential, contemporary evidence indicates a more complex interplay between presynaptic striatal dopamine dysregulation and glutamatergic dysfunction, particularly involving NMDA receptor hypofunction within hippocampal–limbic circuits.^6,7^ These interacting abnormalities are increasingly understood to disrupt large-scale cortico–striato–thalamo–cortical networks that support salience processing, cognition, and affect regulation. Such circuit-level models better account for the diversity of clinical phenotypes, the persistence of negative and cognitive symptoms, and the substantial proportion of individuals who exhibit treatment-resistant illness.

Advances in neuroimaging over the past decade, including structural MRI, diffusion-weighted imaging, functional MRI, positron emission tomography (PET) and magnetic resonance spectroscopy (MRS), have provided increasingly detailed characterisation of these distributed abnormalities. More recent developments, including normative modelling frameworks, connectome-based predictive approaches, and multimodal data fusion, now enable investigation of individual deviation patterns and illness trajectories rather than reliance solely on group-averaged contrasts. These methodological shifts raise important questions regarding whether schizophrenia should be conceptualised not as a static neurodegenerative condition, but as a developmentally anchored disorder of dynamic network instability.

This narrative review synthesises neuroimaging evidence published primarily from 2020 onward to examine how contemporary structural, functional, and molecular findings converge upon systems-level models of schizophrenia. Particular attention is given to heterogeneity, developmental timing, circuit imbalance, and emerging computational approaches that aim to inform prognostic and precision-stratification frameworks.

We argue that current evidence supports conceptualising schizophrenia as a dynamically evolving disorder of distributed cortico–striato–thalamo–cortical circuits. Within these parallel fronto-striatal loops, heterogeneous structural deviations across cortical, striatal, and thalamic nodes disrupt partially dissociable cognitive and limbic pathways, contributing to instability across large-scale functional networks, particularly the salience, executive, and default-mode systems. Dopaminergic and glutamatergic dysregulation are proposed to modulate these circuits through effects on synaptic gain and excitation–inhibition (E/I) balance. Framing schizophrenia in this manner provides a coherent account of symptom heterogeneity while offering a foundation for longitudinal monitoring and precision intervention strategies.

### Populations and terminology

Throughout this review we use the following terminology consistently. ‘Schizophrenia’ refers to participants meeting DSM-5 or ICD-11 diagnostic criteria for the disorder. ‘First-episode’ encompasses schizophrenia, schizoaffective disorder, and other primary psychotic disorders within the first year of treatment. ‘Psychosis-spectrum’ includes affective and non-affective psychoses. ‘Clinical high risk’ (CHR) and ‘genetic high risk’ (gHR) refer to individuals at increased risk of psychosis on attenuated-symptom or familial grounds respectively. ‘Antipsychotic-naïve’ or ‘untreated’ individuals are those who have never received pharmacological treatment, and ‘treatment-resistant schizophrenia’ (TRS) is defined per Howes et al. 2017 criteria.^8^ Transdiagnostic findings spanning schizophrenia, bipolar disorder, and other conditions are signposted explicitly. Throughout, we flag the specific population to which each finding applies, to avoid overgeneralisation across stages of illness.

### Contribution of this review

Several recent reviews have synthesised neuroimaging findings in schizophrenia from complementary angles. Dabiri and colleagues^9^ provided a broad multimodal modality-by-modality update spanning CT, MRI, fMRI, MRS, PET, and MEG. Tuovinen and Hofer^10^ focused specifically on resting-state functional connectivity as a candidate biomarker of treatment response and resistance. Howes, Bukala and Beck^11^ integrated synaptic and pharmacological imaging evidence within a neurochemistry-to-circuits framework. Idowu and colleagues^12^ summarised the diagnostic and predictive performance of machine-learning approaches across imaging modalities. Most directly comparable, Magioncalda, Yadav and Martino,^13^ in an umbrella review of fifty neuroimaging meta-analyses, mapped a spatiotemporal pattern of brain alterations across prodromal, early, and chronic stages and proposed a conceptual framework linking these patterns to symptom dimensions.

The present review differs from these in three respects. First, it foregrounds the conceptual reframing of schizophrenia from a focal or single-neurotransmitter disorder to a disorder of dynamic network instability across cortico–striato–thalamo–cortical circuits and uses imaging evidence to test that frame rather than to summarise pooled effect sizes. Second, it deliberately integrates emerging individual-level approaches (normative modelling, connectome-based predictive modelling, multimodal fusion, and ultra-low-field MRI) alongside the more mature group-average literature, an integration that is necessarily out of scope for an umbrella review restricted to evidence already meta-analysed. Third, it treats heterogeneity and illness stage as central organising problems rather than as residual sources of noise. Table 1 summarises how the focus of the present review differs from these recent reviews.

**Table 1.** Comparison of the present review with five recent neuroimaging reviews in schizophrenia.

Review	Primary focus	Modalities covered	Population scope	Conceptual framing	Coverage of individual-level/computational methods
Dabiri et al. (2022)	Broad multimodal modality-by-modality update	CT, sMRI, fMRI, MRS, PET, MEG, DTI	Schizophrenia (treated and untreated)	Region-based; clinical-radiological	Limited
Tuovinen & Hofer (2023)	Functional connectivity biomarkers of treatment response and resistance	rs-fMRI	Schizophrenia, with emphasis on TRS/UTRS	Dysconnection hypothesis	Moderate
Howes, Bukala & Beck (2024)	Neurochemistry-to-circuits synthesis with treatment implications	PET, MRS, sMRI (synaptic measures)	Prodromal, FEP, chronic schizophrenia	Synaptic/neurochemical pathophysiology	Limited
Idowu et al. (2025)	Diagnostic and predictive performance of ML models	Multimodal (predominantly t-fMRI in meta-analytic pool)	Schizophrenia	Predictive/classification	Extensive (ML focus)
Magioncalda, Yadav & Martino (2025)	Umbrella review of meta-analyses; spatiotemporal model of brain alterations	Grey matter, white matter, intrinsic activity, task fMRI	Prodromal, early and chronic schizophrenia	Spatiotemporal pathophysiology/psychopathology	Limited (constrained by meta-analytic availability)
Present review	Heterogeneity, illness stage, and dynamic network instability	sMRI, dMRI, fMRI, PET, MRS, ULF-MRI, computational	Schizophrenia, FEP, CHR, gHR, transdiagnostic	Dynamic CSTC network instability	Integrated throughout (normative & predictive modelling, multimodal fusion)

## Methods

This narrative review was prepared in accordance with the Scale for the Assessment of Narrative Review Articles (SANRA).^14^ We searched PubMed, Embase, Web of Science, and Scopus for English-language articles published between 1 January 2020 and 31 January 2026, using combinations of the terms ‘schizophrenia’, ‘psychosis’, ‘first-episode psychosis’, and ‘clinical high risk’, paired with modality-specific terms (‘structural MRI’, ‘diffusion MRI’, ‘functional MRI’, ‘positron emission tomography’, ‘magnetic resonance spectroscopy’, ‘ultra-low-field MRI’, ‘normative modelling’, ‘machine learning’, ‘predictive modelling’, ‘multimodal’). Reference lists of identified articles and recent reviews were hand-searched to capture additional relevant studies. Priority was given, in order, to (i) meta-analyses; (ii) large multi-site consortia (including ENIGMA, B-SNIP, AMP-SCZ, and HCP-EP); (iii) longitudinal studies; (iv) multimodal studies; and (v) recent computational work. Foundational older studies (notably Friston et al. 2016 on dysconnection and Oh et al. 2009 on thalamo-frontal tractography) were retained where they remain landmark contributions. Selection was performed by WP with disagreements resolved in discussion with SS. We did not perform a quantitative meta-analysis; this is a thesis-driven narrative synthesis intended to develop a circuit-level conceptual model rather than to estimate pooled effects.

## New insights across imaging modalities

### Structural MRI

Structural magnetic resonance imaging (MRI) has provided some of the most consistent evidence that schizophrenia is associated with measurable brain alterations. Although no single anatomical feature is diagnostic, convergent findings demonstrate subtle yet widespread macrostructural differences, particularly within frontal, temporal, limbic, and thalamic regions.^15^ These distributed alterations have contributed to a gradual shift away from region-specific deficit models toward a systems-level understanding of schizophrenia.

#### Group-level macrostructural alterations

Among the most robust findings are ventricular enlargement and modest reductions in grey matter volume.^16^ Importantly, such alterations are detectable in first-episode and drug-naïve patients, indicating that structural divergence cannot be explained solely by chronic illness or medication exposure. Meta-analytic evidence reinforces this developmental framing. Yang et al.,^17^ analysing 29 studies, reported reduced grey matter volume across frontal, parietal, occipital, and thalamic regions alongside enlarged lateral ventricles. Childhood-onset schizophrenia showed particularly marked reductions in total brain and frontal volumes, whereas first-episode drug-naïve patients exhibited early changes in frontal and thalamic structures. These findings suggest that macrostructural alterations may reflect atypical neurodevelopmental trajectories rather than progressive tissue loss alone.

Beyond global volumetric change, recent large-scale work has refined thalamic involvement at the level of specific nuclei. In the multi-site B-SNIP consortium (n = 2160), mediodorsal (MD) thalamic volumes were consistently reduced across psychosis-spectrum diagnoses relative to controls, with small but reliable effect sizes after correction. In contrast, pulvinar (Pu) volumes did not differ from controls at the aggregate psychosis level, although between-group differences emerged, indicating that thalamic alterations are nucleus-specific rather than uniform. Notably, when participants were stratified using biologically defined Biotypes derived from unsupervised clustering of neurophysiological features,^18^ MD reductions were most pronounced in BT1, which differed from both controls and other Biotypes. This pattern suggests that thalamic structural abnormalities may align more closely with biologically informed subgrouping than with traditional DSM categories.

#### Structural heterogeneity and variability

A second defining feature of structural pathology is heterogeneity. Jiang et al.,^19^ examining over 3,000 individuals, demonstrated significantly greater inter-individual variability in grey matter volume among patients, particularly during early illness. Dispersion was most pronounced in frontotemporal cortex and thalamus in first-episode patients, whereas hippocampal and caudate variability became more evident in chronic stages. Rather than supporting fixed anatomical subtypes, these findings suggest that early illness may involve divergent or unstable neurobiological trajectories that partially converge over time. Structural variability therefore appears dynamic, not static.

#### Normative modelling and developmental trajectories

This reconceptualisation has been further strengthened by normative modelling approaches, which quantify individual deviations from population-expected brain trajectories. While traditional case-control comparisons show widespread cortical thinning and white matter reductions, normative analyses reveal that the anatomical loci of infra-normal deviations are highly inconsistent across individuals, with no single region characterising the majority of patients.^20^ Instead, most individuals show at least one extreme deviation, but its location varies considerably. Importantly, deviation burden correlates with polygenic risk, suggesting that genetic liability manifests as distributed and individually expressed anatomical divergence.

Longitudinal normative studies extend this perspective. Cortical deviations in first-episode psychosis attenuate over time alongside symptom improvement, challenging simplistic neurodegenerative interpretations.^21^ Similarly, extreme regional deviations identified at illness onset predict subsequent negative symptom severity and long-term functional outcome.^22^ In individuals at clinical high risk for psychosis, most macrostructural measures fall within normative ranges,^23^ underscoring that gross morphometric abnormalities alone are insufficient to account for transition risk.

Contemporary structural MRI research supports a model in which schizophrenia reflects developmentally mediated circuit divergence rather than uniform or progressive tissue loss. Ventricular enlargement and cortical thinning remain reliable group-level findings, yet emerging evidence emphasises that macrostructural alterations are heterogeneous, individually expressed, and dynamically related to illness stage and outcome. Normative modelling frameworks further indicate that these structural deviations are better conceptualised as graded departures from population trajectories, with prognostic but not deterministic significance. However, macrostructural imaging captures only part of the picture. Cortical thinning and volumetric reductions do not directly reveal how white matter pathways are organised or how distributed regions communicate. To understand how anatomically dispersed deviations give rise to circuit-level imbalance, it is necessary to examine the microstructural integrity of white matter tracts that support large-scale cortical–subcortical interaction. Diffusion-weighted imaging therefore provides a critical bridge between macrostructural divergence and systems-level dysconnectivity.

### Diffusion weighted imaging

**Figure 1.**
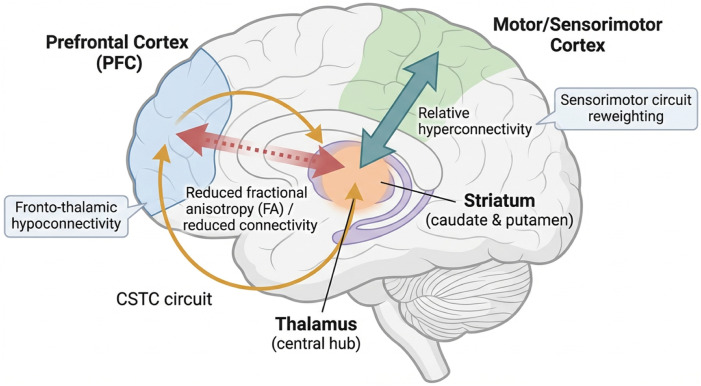
Conceptual schematic illustrating diffusion MRI findings in schizophrenia. Studies consistently report reduced fractional anisotropy and connectivity within fronto-thalamic pathways alongside relative increases in thalamo-sensorimotor connectivity, suggesting selective reweighting of cortico–striato–thalamo–cortical (CSTC) circuits rather than uniform white-matter degeneration. This figure is a conceptual schematic. It is not drawn to anatomical scale, and the relative emphasis on circuits and findings does not represent a weighted quantitative synthesis of the underlying evidence.

Diffusion-weighted imaging (DWI) has substantially advanced the dysconnectivity framework of schizophrenia^24^ by revealing microstructural alterations in white matter pathways that support communication between distributed brain regions. Unlike structural MRI, which captures macroscopic volume differences, diffusion imaging provides insight into the integrity of fibre tracts that link cortical and subcortical systems Figure 1.

#### Fronto-thalamic and thalamocortical circuit imbalance

Early tractography studies identified reduced fractional anisotropy (FA)—a diffusion-derived measure reflecting the directional coherence of water diffusion and often interpreted as an index of white matter microstructural organisation—alongside increased mean diffusivity within thalamo-frontal pathways in individuals with chronic schizophrenia, indicating compromised microstructural organisation along functionally relevant fronto-thalamic tracts.^25^ Importantly, similar reductions in thalamo-prefrontal connectivity have been observed in unaffected siblings of patients,^26^ suggesting that fronto-thalamic hypoconnectivity may reflect familial vulnerability rather than being solely a consequence of illness chronicity. In contrast, the same study found increased thalamo-motor connectivity in both patients and siblings, supporting a model of circuit imbalance rather than uniform white matter degeneration. Together, these findings suggest that schizophrenia involves selective disruption and reweighting of thalamocortical pathways.

More recent work has moved beyond tract-level averages to examine subregional specificity within the thalamus itself. Using advanced diffusion modelling, Alemán-Gómez et al.^27^ identified widespread thalamic microstructural abnormalities in early psychosis, including reduced intra-thalamic fibre integrity and increased extracellular diffusion metrics. These alterations were not uniform, with mediodorsal and pulvinar subregions showing distinct associations with global functioning and negative symptom severity. Such findings extend earlier volumetric evidence of thalamic involvement^28^ by demonstrating that microstructural disruption varies across subnuclei and relates to clinically meaningful dimensions. Given the thalamus’ central role within cortico–striato–thalamo–cortical circuits, these structural disturbances may represent one anatomical substrate through which disrupted dopamine–glutamate interactions destabilise distributed network communication.^29^

#### Developmental trajectories of white matter connectivity

Developmental investigations further refine this picture. In youth with psychosis-spectrum symptoms, thalamocortical tracts show reduced FA and altered maturation trajectories, including attenuated developmental increases during childhood and atypical shifts during adolescence.^30^ Similarly, adolescents with early-onset schizophrenia exhibit decreased dorsolateral prefrontal–thalamic connectivity alongside increased sensorimotor-thalamic coupling, with additional medial prefrontal hyperconnectivity not typically observed in adult-onset illness.^31^ These findings suggest that thalamocortical imbalance emerges during critical neurodevelopmental windows and may vary according to illness stage, reinforcing a model of disrupted circuit maturation rather than static white matter loss.

#### Network topology and connectomics

Diffusion connectomics extends these tract-specific observations to whole-brain network organisation. Graph theoretical analyses reveal reduced clustering coefficients and increased characteristic path length in schizophrenia, indicating diminished network segregation and reduced global efficiency.^32^ Structural disconnections within default mode network pathways appear more pronounced in schizophrenia relative to other major psychiatric disorders, and specific interhemispheric anterior cingulate–medial frontal connections correlate with positive symptom severity. These results demonstrate that local microstructural alterations scale upward to produce measurable topological inefficiency across large-scale networks.

#### Microenvironmental and free-water alterations

Emerging diffusion approaches also highlight alterations in the extracellular microenvironment. Free-water imaging, which separates extracellular diffusion from tissue-specific anisotropy, reveals elevated grey matter free water at illness onset in first-episode psychosis.^33,34^ Increased free water appears stable over 12 months and correlates with negative symptom severity, arguing against progressive neurodegeneration and instead supporting early microenvironmental disruption. Moreover, reduced prefrontal glutathione levels have been associated with elevated free water, linking oxidative stress and neuroimmune processes to diffusion abnormalities.^34^ These findings suggest that diffusion metrics capture not only axonal organisation but also alterations in the extracellular milieu that may interact with glutamatergic and inflammatory mechanisms.

Clinical heterogeneity is likewise reflected in white matter variation. Clinical heterogeneity is likewise reflected in white matter variation. Individuals exhibiting persistent primary negative symptoms (a phenotype sometimes described as a deficit presentation) show more pronounced FA reductions within thalamic radiations and association fibres compared with other patients, with structural compromise correlating with cognitive impairment.^35^ Such subtype-specific patterns reinforce the view that white matter abnormalities map onto symptom dimensions rather than constituting a uniform anatomical signature.

Overall, contemporary diffusion MRI research reinforces a model of selective circuit imbalance emerging during neurodevelopment rather than uniform white matter degeneration. Patterns of fronto-thalamic hypoconnectivity, relative sensorimotor hyperconnectivity, disrupted network topology, and altered extracellular microstructure suggest selective destabilisation of large-scale communication pathways rather than global white matter degeneration. These microstructural alterations provide a plausible anatomical substrate through which functional dysconnectivity may emerge and intersect with dopaminergic and glutamatergic abnormalities explored in subsequent sections.

### Functional MRI

**Figure 2.**
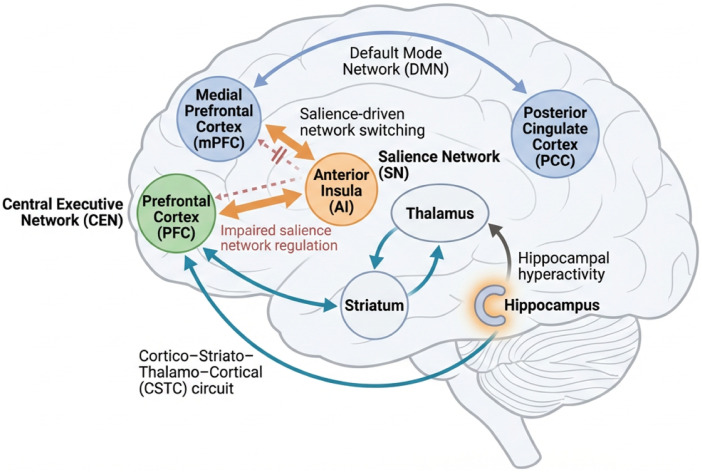
Conceptual schematic summarising functional MRI findings in schizophrenia. Resting-state studies demonstrate dysconnectivity within cortico–striato–thalamo–cortical circuits alongside impaired salience network regulation of default mode and executive networks. Hippocampal hyperactivity further contributes to instability of large-scale network interactions. This figure is a conceptual schematic. It is not drawn to anatomical scale, and the relative emphasis on circuits and findings does not represent a weighted quantitative synthesis of the underlying evidence.

While structural and diffusion imaging delineate the anatomical substrate of dysconnectivity, functional MRI (fMRI) provides insight into how these circuits operate dynamically in vivo. Resting-state functional connectivity studies increasingly conceptualise schizophrenia as a disorder of large-scale network integration rather than focal dysfunction. Consistent with diffusion evidence of thalamocortical imbalance, functional studies reveal altered connectivity within cortico–striato–thalamo–cortical (CSTC) circuits, with both hyper- and hypoconnectivity patterns depending on network context and illness stage Figure 2.

#### Thalamocortical and CSTC dysconnectivity

Subregional thalamic analyses demonstrate increased connectivity between specific thalamic nuclei and sensorimotor, visual, and medial frontal cortices, alongside reduced connectivity with prefrontal regulatory regions, even in first-episode and drug-naïve samples.^36,37^ Similarly, first-episode schizophrenia is characterised by salience network (SN) hyperconnectivity with prefrontal and cerebellar regions and concurrent hypoconnectivity within CSTC subcircuits.^38^ These patterns parallel microstructural evidence of fronto-thalamic hypoconnectivity and relative sensorimotor hyperconnectivity, suggesting that structural circuit imbalance manifests functionally as disrupted network coordination.

#### Large-scale network regulation and the triple-network model

Large-scale network mapping further supports a staged model of dysconnectivity. Xu et al.^39^ demonstrated that structural abnormalities in clinical high-risk (cHR) and genetic high-risk (gHR) individuals map onto distinct functional networks, with cHR alterations predominantly involving the frontoparietal network and gHR alterations implicating subcortical networks. In contrast, first-episode and chronic schizophrenia showed convergent patterns involving somatomotor, ventral attention, and subcortical networks, suggesting that heterogeneous vulnerability pathways may coalesce into more widespread and trait-like network abnormalities following illness onset. This pattern aligns with structural heterogeneity findings and supports a progression from network-specific vulnerability to distributed systems disruption following illness onset.

Beyond pairwise connectivity, contemporary models emphasise large-scale network regulation. The triple-network framework posits that the salience network orchestrates switching between the default mode network (DMN) and central executive network (CEN), enabling adaptive cognitive control. In first-episode psychosis and individuals at clinical high risk, reduced SN–DMN and SN–CEN connectivity indicates early disruption of this regulatory mechanism.^40^ Extending this, stochastic dynamic causal modelling reveals that in healthy individuals the SN exerts directional control over DMN and CEN activity, whereas in schizophrenia this controlling influence is markedly impaired.^41^ Such findings suggest that aberrant salience attribution may reflect a failure of network-level gating rather than isolated regional abnormalities, offering a systems-level account of psychotic symptom formation.

#### Hippocampal hyperactivity and glutamatergic modulation

Hippocampal dysfunction represents a critical node within this dysregulated architecture. Resting-state studies demonstrate hyperactivity, particularly within the caudal hippocampus, accompanied by increased connectivity to the thalamus, putamen, middle frontal gyrus, and parietal cortex.^42^ Hippocampal hyperactivity correlates with symptom severity, reinforcing its clinical significance. Complementary evidence from drug-naïve first-episode cohorts shows hippocampal hypoconnectivity with anterior DMN regions alongside hyperconnectivity with lateral occipital cortex, indicating network-specific redistribution rather than uniform increases in connectivity.^43^ This study also found hippocampal glutamate levels to predict functional connectivity strength in healthy individuals, a relationship which is reversed in first-episode psychosis. This disruption of glutamatergic modulation suggests that altered excitation–inhibition balance may impair the hippocampus’ capacity to regulate distributed networks, providing a mechanistic bridge between molecular and systems-level dysfunction.

#### Dynamic and effective connectivity

Dynamic functional connectivity analyses further reveal increased temporal instability in thalamic and large-scale network interactions.^37^ Rather than occupying stable connectivity states, individuals with schizophrenia exhibit altered transition dynamics and greater fluctuations between network configurations, consistent with developmental models of unstable circuit maturation. Effective connectivity studies similarly demonstrate disrupted directional influences from limbic regions to the thalamus, highlighting impaired bottom-up modulation within core regulatory pathways.^44^

Contemporary fMRI evidence portrays schizophrenia as a disorder of impaired network regulation characterised by thalamocortical imbalance, disrupted salience-driven switching, hippocampal hyperactivity, altered glutamatergic modulation, and developmental convergence of network abnormalities at illness onset. These dynamic disturbances likely represent the functional expression of the microstructural alterations described in diffusion imaging and provide a systems-level framework through which dopaminergic and glutamatergic dysregulation may converge to generate psychotic and cognitive symptoms. Molecular imaging approaches offer a critical next step in directly interrogating these neurochemical mechanisms and clarifying their relationship to network dysfunction.

### Molecular imaging: Positron emission tomography (PET) and magnetic resonance spectroscopy (MRS)

While structural, diffusion, and functional MRI delineate the anatomical and systems-level architecture of dysconnectivity, positron emission tomography (PET) and magnetic resonance spectroscopy (MRS) allow direct interrogation of the neurochemical mechanisms hypothesised to underlie these network abnormalities. Contemporary molecular imaging evidence converges on two interacting systems: presynaptic dopaminergic dysregulation and glutamatergic excitation–inhibition (E/I) imbalance. Crucially, both systems demonstrate substantial heterogeneity across symptom dimensions and illness stages.

#### Presynaptic dopaminergic dysregulation

The revised dopamine hypothesis proposes increased presynaptic dopamine synthesis and release within the striatum, particularly the associative subdivision, rather than uniform postsynaptic D_2_ receptor excess. Consistent with this, first-episode psychosis cohorts demonstrate elevated striatal dopamine synthesis capacity compared to controls across psychotic diagnoses (transdiagnostic across schizophrenia, delusional disorder, and other psychotic disorders), supporting a dimensional rather than diagnostically fixed model of dopaminergic dysregulation.^45^ Elevation is most prominent in the associative striatum, a subdivision that receives dense cortico-striatal projections from prefrontal cortical regions and is involved in salience processing and cognitive control.

Recent work refines this model further. Jauhar et al.^46^ showed that dopamine synthesis capacity varies according to affective state within psychotic disorders: higher associative striatal synthesis correlated with greater positive symptom severity, while limbic striatal differences distinguished depressive from manic presentations. Dopaminergic dysregulation therefore appears dimensionally expressed rather than diagnostically fixed. Importantly, dopaminergic elevation is not universal. In two independent cohorts of medication-free individuals with schizophrenia, Eisenberg et al.^47^ observed no overall group increase in dopamine synthesis capacity. Instead, lower striatal synthesis was robustly associated with greater negative symptom burden. These findings challenge the assumption that hyperdopaminergia is a defining feature in all cases and underscore biologically meaningful heterogeneity.

#### Dopamine and treatment response

Further nuance emerges when considering treatment response. In antipsychotic-naïve first-episode psychosis, baseline striatal decarboxylation rates (k3), reflecting presynaptic dopamine synthesis capacity, were associated with positive symptom severity and predicted symptomatic improvement following D2 partial agonist treatment.^48^ Multimodal PET–DTI work further demonstrates that in treatment-responsive patients, reduced frontostriatal structural connectivity correlates with elevated associative striatal dopamine synthesis, whereas this coupling is absent in treatment-resistant schizophrenia.^49^ Together, these findings support a circuit-regulated model in which impaired prefrontal control may disinhibit striatal dopamine signalling in some individuals, while alternative neurobiological mechanisms may predominate in treatment-resistant forms.

Contemporary PET evidence suggests that striatal dopamine abnormalities are symptom-linked, subregion-specific, and modulated by cortical circuitry rather than representing a uniform or static abnormality.

#### Glutamatergic imbalance and excitation–inhibition disruption

Parallel to dopaminergic findings, MRS studies implicate glutamatergic dysregulation and E/I imbalance. A large meta-analysis of 134 studies (7,993 patients with schizophrenia-spectrum disorders and 8,744 controls) reported elevated combined glutamate and glutamine (Glx) levels in the basal ganglia and hippocampus, particularly in unmedicated and early-stage cohorts, alongside reduced GABA in the mid-cingulate cortex.^50^Treatment-resistant schizophrenia showed distinct elevations in medial cingulate glutamate, again suggesting subtype-specific neurochemical profiles.

In drug-naïve first-episode psychosis, hippocampal glutamate levels are associated with duration of untreated psychosis (DUP), with higher Glx observed in individuals with prolonged DUP and concurrent reductions in hippocampal subfield volumes.^51^ These findings highlight the potential clinical importance of early intervention. However, longitudinal data complicate a simple excitotoxicity model. Nelson et al.^52^ reported progressive hippocampal subfield volume loss over 16 weeks of treatment without baseline glutamate differences or glutamate–volume coupling, suggesting that structural progression is unlikely to be driven solely by elevated glutamate concentrations.

#### Neurochemical circuit coupling

Importantly, multimodal evidence indicates altered coupling between glutamate levels and functional connectivity. In healthy individuals, hippocampal glutamate positively predicts connectivity strength within regulatory networks, whereas in first-episode psychosis this relationship is reversed.^43^ This decoupling suggests that glutamatergic imbalance may impair network modulation and regulatory capacity rather than simply increasing excitatory tone.

Taken together, molecular imaging findings support a multi-level framework in which developmental circuit vulnerability contributes to hippocampal and cortical dysregulation, altering glutamatergic modulation and destabilising large-scale networks. In some individuals, reduced prefrontal control over striatal circuits may facilitate presynaptic dopamine excess within associative striatum, particularly in relation to positive symptoms. However, variation across mood states, negative symptoms, and treatment response indicates that dopaminergic and glutamatergic abnormalities are dynamically expressed across individuals and illness stages.

Rather than representing competing hypotheses, dopamine and glutamate models appear complementary within a circuit-based framework. PET and MRS findings provide mechanistic constraints on network-level dysconnectivity, suggesting that neurochemical dysregulation is embedded within, and partially regulated by, distributed cortico–striatal–thalamo–limbic systems. Future longitudinal and multimodal approaches will be critical for clarifying how neurochemical alterations interact with structural and functional network trajectories over time.

Before turning to computational approaches, it is useful to consolidate the evidence reviewed so far. Table 2 maps each imaging modality onto its principal findings, the strength of the underlying evidence base, the biological interpretation, the principal limitations, and the current clinical relevance. Two patterns recur across modalities: robust group-level effects coexist with substantial inter-individual heterogeneity, and few findings have yet reached the threshold of clinically actionable biomarkers. Both observations motivate the individual-level, computational methods considered next.

**Table 2.** Synthesis of imaging modalities reviewed: principal findings, evidence base, biological interpretation, limitations, and clinical relevance.

Modality	Key findings	Evidence base	Biological interpretation	Principal limitations	Clinical relevance
Structural MRI	Distributed cortical thinning, ventricular enlargement, mediodorsal thalamic volume reduction; high inter-individual variability	Meta-analytic (Yang et al. 2025,n=29 studies); B-SNIP n=2,160; Jiang et al. 2025 n>3,000	Developmentally anchored circuit divergence rather than uniform tissue loss	Cross-sectional designs; medication confound; small effect sizes	Group-leveldifferences; not yet diagnostic
Diffusion MRI	Reduced FA in fronto-thalamic pathways; relative thalamo-sensorimotorhyperconnectivity; elevated grey-matter free water	Replicated tract-based studies; dispersion observed in unaffected siblings	Selective circuit reweighting; extracellular microenvironmental disruption	Metric heterogeneity; scanner variability; non-specific to schizophrenia	Research only
Functional MRI	Salience–default–executive network dysconnectivity; thalamocortical imbalance; hippocampal hyperactivity; impaired triple-network gating	Multiple medium-to-large studies; mixed meta-analytic findings	Dynamic network instability; impaired salience-driven switching	Motion; task heterogeneity; reproducibility concerns	Research only; some prognostic signal in CPM
PET	Elevated presynaptic associative-striatal dopamine synthesis capacity; symptom-linked, not universal	Multiple replicated cohorts; recent normative modelling (Giacomel et al. 2025)	Subregion-specific hyperdopaminergia; partly cortically regulated	Small samples; ligand availability; cost; not all patients elevated	Stratification potential, especially in TRS prediction
MRS	Elevated basal-ganglia and hippocampal Glx; reduced mid-cingulate GABA; medial-cingulate Glx elevation in TRS	Meta-analysis (Nakahara et al. 2022, n=7,993)	Excitation–inhibition imbalance, possibly stage-dependent	Voxel placement; quantification methods	Research only
Normative modelling	Individual-level deviation maps (sMRI, dMRI, PET); deviation burden correlates with polygenic risk; predicts treatment response (AUC 0.77–0.83)	Lv et al. 2021; Berthet et al. 2024 (10-yr follow-up); Giacomel et al. 2025	Heterogeneity captured at single-subject level	Reference-cohortrepresentativeness; out-of-sample validation	Pre-clinical proof of concept for stratification
Predictive modelling/multimodal	CPM predicts symptom dimensions and antipsychotic response; multimodal fusion improves transition prediction (AUC 0.73)	Cao et al. 2023 (replicated); Reinen et al. 2024; Porter et al. 2023 meta-analysis	Distributed network signatures rather than focal markers	Within-dataset cross-validation; limited external validation	Promising but not clinically deployed
Ultra-low-field MRI	Feasibility of clinical-scale deployment; super-resolution improves volumetric correspondence with 3T	Single-site (Cooper et al. 2024); not yet validated in psychosis cohorts	Hardware-driven scalability; cost and tolerability advantages	Limited resolution; cortical-thickness reliability	Potential for global access; not validated for psychosis

## Computational approaches to heterogeneity and prediction in schizophrenia

**Figure 3.**
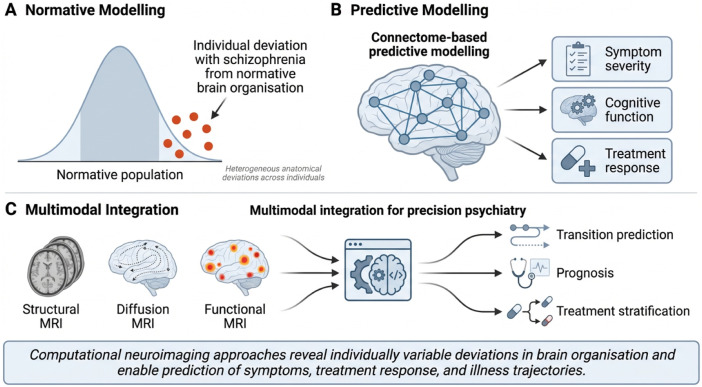
Computational neuroimaging approaches in schizophrenia. (A) Normative modelling quantifies individual deviations from population brain organisation, highlighting marked heterogeneity across patients. (B) Connectome-based predictive modelling uses distributed connectivity patterns to estimate symptom severity, cognitive function, and treatment response. (C) Multimodal integration combines structural, diffusion, and functional imaging to improve prediction of illness trajectories and enable precision stratification. This figure is a conceptual schematic. It is not drawn to anatomical scale, and the relative emphasis on circuits and findings does not represent a weighted quantitative synthesis of the underlying evidence.

Recent advances in computational neuroimaging have reshaped how schizophrenia is conceptualised. Rather than focusing solely on group-averaged abnormalities, the field increasingly emphasises individualised deviation mapping, predictive modelling, and multimodal integration. These approaches directly address a central theme emerging across imaging modalities: marked inter-individual heterogeneity within distributed circuit abnormalities Figure 3.

### Normative modelling across modalities

Normative modelling provides a principled framework for quantifying how an individual’s brain deviates from population-expected trajectories. In a landmark cross-sectional study, Lv et al.^20^ showed that although patients with schizophrenia exhibit widespread group-level reductions in cortical thickness and white matter fractional anisotropy, no single region displayed infra-normal deviations in more than 15–20% of individuals. Instead, 79% of patients demonstrated at least one extreme deviation, but the anatomical loci varied substantially across individuals. Importantly, deviation burden correlated with polygenic risk, suggesting that genetic liability manifests as distributed and individually specific structural divergence rather than a uniform anatomical signature.

Longitudinal data extend this framework. Berthet et al.,^21^ in a 10-year follow-up study of first-episode psychosis, found that cortical deviations attenuated over time alongside symptom reduction. This pattern challenges a simple neurodegenerative account and instead supports a dynamically evolving model in which early deviations may stabilise or partially normalise. Complementing this, Muñoz-Caracuel et al.^53^ demonstrated that baseline deviations in superior temporal and inferior frontal regions predicted long-term symptom and functional trajectories across 1, 3, and 10-year follow-up. Normative deviation patterns therefore appear to carry prognostic value, indexing developmental vulnerability and modulating illness course rather than merely distinguishing cases from controls.

Normative modelling has also been extended to molecular imaging. Giacomel et al.^54^ applied this framework to dopaminergic PET measures and observed a threefold increase in extreme deviations among patients with psychosis relative to controls, again with minimal spatial overlap across individuals. Importantly, normative-referenced striatal dopamine synthesis predicted treatment response (AUC 0.77–0.83), illustrating that molecular heterogeneity can be quantified and leveraged for precision stratification. These findings reinforce the view that dopaminergic dysregulation is variably expressed within a shared circuit framework rather than uniformly elevated.

### Predictive modelling of symptoms and treatment response

Beyond deviation mapping, connectome-based predictive modelling (CPM) has been applied to estimate symptom severity from distributed connectivity patterns. In a large multi-site study, Ou et al.^55^ used CPM with cross-validation to predict negative, positive, affective, and cognitive symptom dimensions from resting-state connectivity, implicating interactions among motor/sensory, frontoparietal, default mode, and salience networks. Foster et al.^56^ similarly applied CPM to identify connectivity patterns predicting symptom profiles in early and chronic psychosis, with stronger model performance in chronic illness, suggesting that network-level symptom signatures may consolidate over time. While these approaches demonstrate the potential of connectivity-based prediction, most studies rely on cross-validation within single datasets, and independent external validation will be necessary to establish clinically robust predictive biomarkers .

Predictive modelling has also demonstrated translational potential in treatment forecasting. Cao et al.^57^ identified a paradigm-independent functional connectome signature that predicted individualised antipsychotic response in first-episode psychosis, with replication in an independent validation cohort. Predictive features involved cerebellar–cortical and higher-order cognitive circuitry, indicating that treatment response reflects distributed systems-level organisation rather than isolated regional abnormalities.

### Multimodal integration and transition prediction

Given the multilevel nature of schizophrenia pathology, multimodal integration represents a logical progression. Reinen et al.^58^ applied multiple kernel learning to structural, diffusion, and functional MRI data in individuals at clinical high risk for psychosis. Multimodal models outperformed unimodal approaches in predicting transition (AUC 0.73), identifying convergent contributions from frontal, cingulate, thalamic, and striatal regions. These findings suggest that cross-modality redundancy and synergy may stabilise predictive models and better capture complex circuit-level vulnerability.

However, methodological caution is warranted. A recent meta-analysis by Porter et al.^59^ found that structural, diffusion, and functional modalities demonstrate broadly similar classification performance for psychosis, with modest effect sizes and substantial heterogeneity across studies. Multimodal approaches did not consistently outperform unimodal models. These findings highlight the need for external validation, harmonisation across sites, and longitudinal prediction rather than cross-sectional case–control classification.

Computational approaches converge on several key insights. First, schizophrenia is characterised by widespread but individually variable deviations from normative brain organisation. Second, these deviations evolve over time, supporting a developmentally anchored yet dynamically modulated circuit disorder. Third, symptom expression and treatment response emerge from distributed network configurations rather than singular regional deficits. Finally, multimodal integration offers incremental gains in prognostic modelling, though rigorous validation remains essential.

Importantly, these computational frameworks do not replace structural, diffusion, functional, or molecular imaging findings. Rather, they provide the analytic infrastructure needed to reconcile heterogeneity with shared circuit-level vulnerability. In doing so, they strengthen a unifying model in which developmentally shaped structural deviations and microstructural alterations converge into large-scale network instability, modulated by interacting dopaminergic and glutamatergic processes. Future work integrating normative modelling, multimodal fusion, and longitudinal trajectory analysis may work toward personalised stratification grounded in systems neuroscience rather than categorical diagnosis.

## Future directions

### Current translational status

Despite considerable progress, neuroimaging findings in schizophrenia remain primarily research tools. No imaging-derived biomarker has yet been validated for use in routine psychiatric practice; meta-analytic findings, even where statistically robust, typically reflect group-level effects with substantial overlap between patients and unaffected individuals at the single-subject level. Promising approaches such as normative modelling, predictive modelling, neuromodulation targeting, and neurofeedback have demonstrated proof of concept in research cohorts but have not yet achieved the levels of generalisability, regulatory approval, or cost-effectiveness required for clinical deployment. We therefore frame the translational implications below as plausible directions of travel for the next decade rather than imminent clinical applications.

### Heterogeneity, longitudinal designs, and harmonisation

Recent large-scale neuroimaging collaborations have begun to clarify the extent of heterogeneity in schizophrenia-related brain alterations. The ENIGMA Clinical High Risk for Psychosis consortium demonstrated that individuals at clinical high risk exhibit substantial divergence in global cortical and subcortical profiles at the individual level despite relatively modest group-level differences.^60^ Only a minority of individuals displayed extreme normative deviations, and these were not strongly linked to transition status. These findings suggest that early neuroanatomical variation reflects distributed developmental divergence rather than a deterministic lesion signature. However, such evidence remains largely cross-sectional. While cross-sectional analyses can reveal heterogeneity across individuals, they cannot determine whether observed deviations represent stable developmental traits, progressive alterations, or compensatory adaptations over time. This limitation is particularly important if schizophrenia is conceptualised not as a static lesion model but as a dynamically evolving disorder of neurodevelopmentally anchored circuits.

Longitudinal approaches begin to address this challenge. Normative modelling across a ten-year follow-up of first-episode psychosis indicates that cortical deviations may attenuate alongside symptom improvement,^21^ challenging simple neurodegenerative accounts of schizophrenia. Complementing this, baseline deviations in superior temporal and inferior frontal regions predict long-term symptom severity and functional outcomes across multiple follow-up intervals.^53^ Together, these findings suggest that early structural divergence may shape illness trajectories without necessarily progressing toward uniform deterioration. Addressing such questions at scale will require large, harmonised longitudinal datasets.

The field is now developing infrastructure to test integrated developmental models. The Accelerating Medicines Partnership Schizophrenia Program (AMP SCZ) has implemented a harmonised, multi-site protocol incorporating structural, resting-state functional, and diffusion MRI across timepoints in the largest clinical high-risk cohort to date.^61^ By quantifying participant, site, and platform variance, this initiative addresses a major barrier to longitudinal inference and enables modelling of how microstructural alterations, network instability, and symptom dimensions co-evolve during transition to psychosis.

Future research should move beyond descriptive change to mechanistic modelling. Dynamic causal modelling suggests diverse electrophysiological and functional abnormalities may converge on reduced pyramidal cell synaptic gain,^62^ providing a plausible bridge between excitation–inhibition imbalance and network instability. Embedding such models within longitudinal designs may clarify whether early synaptic alterations precede or follow circuit-level dysconnectivity.

For clinical translation, harmonisation and generalisability remain essential. Multi-site investigations show scanner-specific harmonisation can achieve stable discrimination across 15 MRI platforms.^63^ Although classification accuracy remains modest, such advances are critical for scalability. Standardised acquisition, harmonised preprocessing, and external validation will be prerequisites for clinically actionable imaging frameworks.

Integrating multimodal imaging with digital phenotyping and longitudinal symptom monitoring represents an additional priority. Network instability likely fluctuates across clinical states, treatment exposure, and environmental stressors. High-frequency behavioural and physiological measures may therefore complement imaging-derived trajectories and better align neural models with lived symptom variability.

### Heterogeneity, longitudinal designs, and harmonisation

A further consideration concerns the accessibility and tolerability of neuroimaging technologies. Any role for imaging in clinical stratification or monitoring will depend on infrastructure that is widely available and tolerable for individuals with severe mental illness, who frequently experience comorbid anxiety, agitation, or cognitive impairment. Emerging ultra-low-field (ULF) MRI systems and head-only or more open scanner designs may help address these barriers by reducing cost, improving portability, and increasing patient tolerability, with particular relevance for low- and middle-income settings and for services where conventional MRI is unavailable. Computational super-resolution techniques may partially mitigate the resolution limitations of ULF scanners; in a recent comparison of 64 mT Hyperfine and 3T MRI acquisitions in young participants, convolutional neural network–based reconstruction substantially improved correspondence for volumetric and surface area estimates, although finer-scale measures such as cortical thickness remained less reliable.^64^ Importantly, no ULF-MRI study has yet been conducted in psychosis cohorts; feasibility for clinical scalability has been demonstrated in principle, but validation in schizophrenia and related disorders remains a prerequisite for any future clinical application.

### Therapeutic implications

Advances in understanding circuit-level pathophysiology may also inform the development of therapeutic strategies that directly target dysregulated neural networks. Non-invasive neuromodulation approaches, including transcranial magnetic stimulation (TMS) and transcranial direct current stimulation (tDCS), are increasingly conceptualised as tools for modulating dysfunctional large-scale brain networks rather than isolated cortical regions.^65^ Complementary psychological approaches aim to modify cognitive processes linked to psychotic symptoms; for example, cognitive bias modification targeting maladaptive interpretations associated with paranoia has demonstrated feasibility and preliminary symptom improvements in randomised trials.^66^ In parallel, neurofeedback paradigms provide a more direct route for circuit modulation. Real-time fMRI neurofeedback has shown that patients with schizophrenia can learn to down-regulate activity within the superior temporal gyrus, a region implicated in auditory hallucinations, with associated changes in functional connectivity and reductions in hallucination severity.^67^ These approaches provide preliminary, proof-of-concept evidence that mechanistic insights from neuroimaging could in future inform the development of interventions combining pharmacological, behavioural, and neuromodulatory strategies; substantial validation in independent cohorts and in routine-care settings will be required before clinical adoption. Emerging precision psychiatry frameworks^68^ similarly emphasise the integration of biological and symptomatic dimensions as a longer-term path toward mechanism-informed stratification.

Taken together, the next phase of schizophrenia research will require coordinated, longitudinal, and multimodal investigations grounded in mechanistic modelling and supported by harmonised infrastructure. Rather than focusing on cross-sectional differences at a single timepoint, future studies must characterise how neural deviations emerge, stabilise, or diverge across developmental stages and illness progression. Such trajectory-based approaches provide the most direct test of whether heterogeneous early alterations ultimately converge into the large-scale network instability that characterises established schizophrenia.

## Limitations

Several limitations should be acknowledged. First, much of the imaging literature in schizophrenia is confounded by medication effects, particularly cumulative antipsychotic exposure, which influences grey-matter volume, white-matter microstructure, and functional connectivity, and which is rarely fully accounted for in cross-sectional designs. Second, illness stage is heterogeneously defined across studies, with first-episode, chronic, and treatment-resistant cohorts often pooled in ways that obscure stage-specific effects; we have flagged the population to which each finding applies, but the underlying primary literature retains this limitation. Third, between-site and between-scanner variability remains a substantial source of noise, even after the application of harmonisation pipelines such as ComBat^69^; recent multi-site work demonstrates that harmonisation can stabilise discrimination across platforms but does not eliminate site-related variance. Fourth, the field continues to face challenges around sample size, demographic representativeness, and replication, and many striking single-study findings have not been independently confirmed. Fifth, this review is itself a narrative, thesis-driven synthesis. Although we prioritised meta-analyses and large multi-site studies and have made our search and selection approach explicit, the selection of evidence is necessarily shaped by the conceptual frame of dynamic network instability, and other defensible framings of the same literature exist.

## Conclusion

Over the past five years, advances in structural, diffusion, functional, molecular, and computational neuroimaging have refined our understanding of schizophrenia. Converging evidence supports a framework in which schizophrenia reflects heterogeneous, developmentally shaped deviations in distributed neural circuits that manifest as large-scale network instability, with dopaminergic and glutamatergic abnormalities expressed in subregion-specific and dynamically modulated ways. Heterogeneity is not noise but a defining feature of the disorder. Significant work nevertheless remains before neuroimaging can contribute meaningfully to personalised psychiatric care: priorities include validation of candidate markers in independent and demographically representative cohorts, demonstration of clinical utility against current standards of care, rigorous handling of medication and illness-stage confounds, and prospective evaluation of stratified-treatment paradigms. Until these are achieved, neuroimaging in schizophrenia is best understood as a maturing research enterprise rather than an imminent clinical tool.

## Appendix

### Abbreviations

#### Abbreviation definition

AMP-SCZ: Accelerating Medicines Partnership Schizophrenia Program
AUC: Area under the curve
B-SNIP: Bipolar-Schizophrenia Network on Intermediate Phenotypes
CEN: Central executive network
CHR: Clinical high risk for psychosis
CPM: Connectome-based predictive modelling
CSTC: Cortico–striato–thalamo–cortical
DMN: Default mode network
DTI: Diffusion tensor imaging
DUP: Duration of untreated psychosis
DWI: Diffusion-weighted imaging
E/I: Excitation–inhibition
ENIGMA: Enhancing Neuro Imaging Genetics through Meta-Analysis consortium
FA: Fractional anisotropy
FEP: First-episode psychosis
fMRI: Functional magnetic resonance imaging
GABA: Gamma-aminobutyric acid
gHR: Genetic high risk for psychosis
Glx: Combined glutamate and glutamine signal
MD: Mediodorsal (thalamic nucleus)
MRI: Magnetic resonance imaging
MRS: Magnetic resonance spectroscopy
NMDA: N-methyl-D-aspartate
PET: Positron emission tomography
Pu: Pulvinar (thalamic nucleus)
rs-fMRI: Resting-state functional MRI
sMRI: Structural MRI
SN: Salience network
TMS: Transcranial magnetic stimulation
TRS: Treatment-resistant schizophrenia
tDCS: Transcranial direct current stimulation
ULF-MRI: Ultra-low-field magnetic resonance imaging.
